# Rare case of torsion of giant ovarian mass post-colonoscopy

**DOI:** 10.1093/jscr/rjab070

**Published:** 2021-03-29

**Authors:** Irene A T Ng, Jolene S M Wong, Jermaine Wong, Claramae S Chia, Chin-Ann J Ong

**Affiliations:** 1 Department of Sarcoma, Peritoneal and Rare Tumours (SPRinT), Division of Surgery and Surgical Oncology, National Cancer Centre Singapore, Singapore; 2 Raffles Institution, Singapore, Singapore; 3 SingHealth Duke-NUS Oncology Academic Clinical Program, Duke-NUS Medical School, Singapore, Singapore; 4 Laboratory of Applied Human Genetics, Division of Medical Sciences, National Cancer Centre Singapore, Singapore; 5 Institute of Molecular and Cell Biology, A^*^STAR Research Entities, Singapore, Singapore

## Abstract

We present an unprecedented case of torsion of a large ovarian cyst following colonoscopy. A 43-year-old female was found to have a 20 × 13 × 19 cm pelviabdominal mass possibly arising from the right ovary. Endoscopic evaluation was performed prior to planned resection of the ovarian mass. The patient experienced progressive lower abdominal pain after the procedure with a computed topography finding of torsion. She underwent exploratory laparotomy, right salpingo-oophorectomy with intra-operative frozen section and omentectomy. Final histology revealed features of benign serous cystadenoma with extensive haemorrhagic infarction in keeping with torsion. To our knowledge, this is the first reported case of torsion of a large ovarian cyst after colonoscopy. We propose a postulated mechanism of this patient’s ovarian torsion and urge clinicians to be cognizant of acute ovarian torsion as a cause of severe abdominal pain following endoscopy.

## INTRODUCTION

Ovarian torsion is a surgical emergency that occurs when there is twisting of the ovarian tissue on its pedicle leading to reduced venous return, stromal oedema, internal haemorrhage and infarction.

Krukenberg tumours are ovarian metastases secondary to gastrointestinal primaries, occurring in ~50% of this cohort [1]. Some physicians perform routine endoscopic evaluation in patients who present with suspicious ovarian masses.

Although complications of colonoscopy are well studied, ovarian torsion following colonoscopy has not been reported.

## CASE REPORT

A 43-year-old female presented with a pelvic mass. Computed tomography (CT) of the chest, abdomen and pelvis showed a well circumscribed 20.7 × 13.4 × 19.0 cm predominantly homogeneous soft tissue density pelviabdominal mass, without definite enhancement, calcification or intralesional fat. The lesion was further characterized with magnetic resonance imaging pelvis, which showed a pelviabdominal cystic mass superior to the retroverted uterus, measuring 21.4 × 18.0 × 11.1 cm, with papillary mural components posteriorly in keeping with an ovarian neoplasm. Cancer antigen 125 (CA-125) was elevated at 144, cancer antigen 19-9 (CA 19-9) was <9.0 and carcinoembryonic antigen (CEA) < 1.8.

The case was discussed in a multi-disciplinary tumour board meeting and decision was made for surgical resection. She underwent an uneventful esophagogastroduodenoscopy and colonoscopy prior to surgery with no significant abnormalities found.

Following endoscopy, the patient developed worsening abdominal pain associated with vomiting. On post-procedural Day 2, she presented to the emergency department with significant tenderness over the right iliac fossa and with guarding. There was a large palpable mass extending above the umbilicus that was tender on palpation.

**Figure 1:**
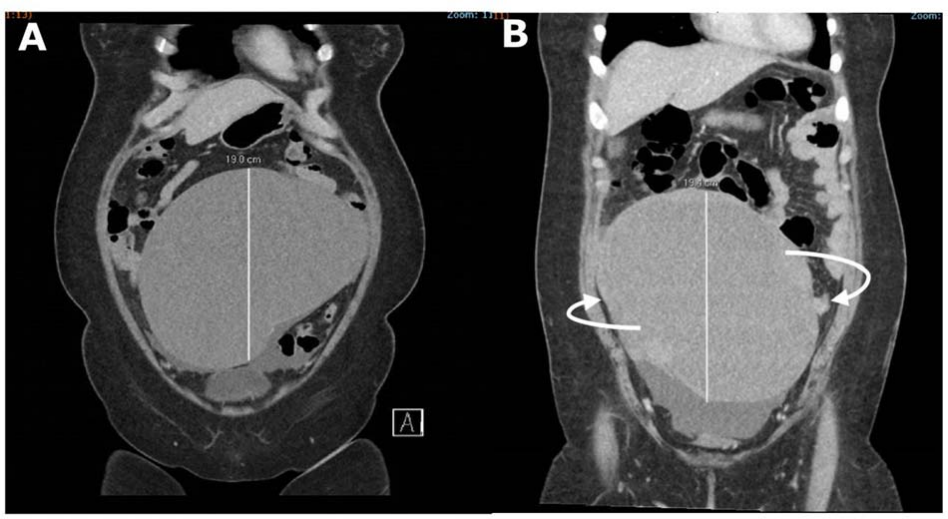
CT abdomen and pelvis (CTAP) showing acute change in orientation of the right ovarian mass before (**A**) and after (**B**) colonoscopy.

An urgent CT abdomen and pelvis (CTAP) revealed a change in orientation of the large cystic adnexal mass with possible signs of intra-tumoural haemorrhage (Fig. 1). Blood investigations found a haemoglobin level of 9.6 g/dL from a baseline of 12.6 g/dL, leukocytosis of 15.5 × 10^9^/L and lactate was slightly elevated at 1.5 mmol/L.

**Figure 2:**
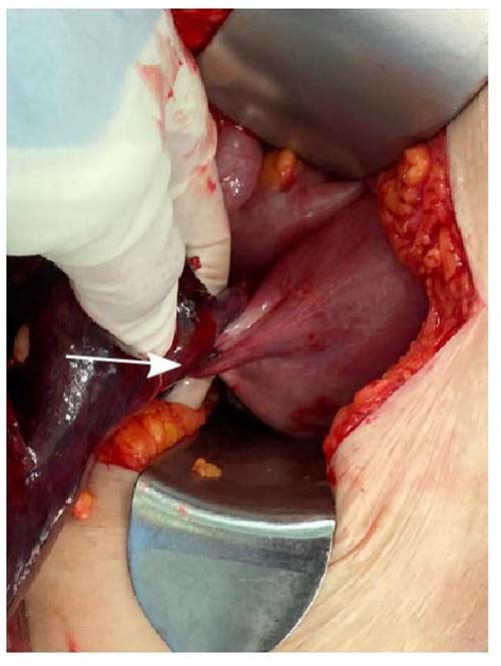
Torsion of right ovarian pedicle seen intra-operatively.

A diagnosis of right ovarian torsion was made and the patient underwent exploratory laparotomy, right salpingo-oophorectomy with intra-operative frozen section and omentectomy. Intra-operatively, torsion of right gonadal vessels was noted (Fig. 2). The right gonadal vessels were taken between ties. The right fallopian tube was ligated. Frozen section from the right salpingo-oophorectomy showed features of benign serous cystadenoma with extensive haemorrhagic infarction in keeping with torsion. There was no evidence of malignant cells. Omentectomy was performed and sent for frozen section, which returned with no evidence of malignancy. The abdominal cavity was washed thoroughly and closed with sutures.

The patient recovered well and was discharged on the fifth post-operative day. The patient was reviewed in clinic 2 weeks following her surgery. She remained well and asymptomatic.

## DISCUSSION

Up to 30% of ovarian masses are secondary to metastases from gastrointestinal, breast or other rare primaries. The upper and lower GI tract accounts for a majority of these Krukenberg tumours. Given the ease of endoscopic evaluation, some centres propose routine evaluation of these sites in women who present with large ovarian masses. Our patient is a pre-menopausal woman with a large (>10 cm) unilateral ovarian mass, associated with elevated CA-125 with symptoms of intermittent constipation. As such, both gastroscope and colonoscopy were necessary to exclude a gastrointestinal primary malignancy prior to definitive surgery for her ovarian tumour.

Although complications of colonoscopy are well studied [2], rarer complications such as mesentery and gallbladder torsion have only been reported as case studies [3–5]. In the reported case of gallbladder torsion following colonoscopy, the patient was found to have a ‘wandering’ gallbladder. It was postulated that air insufflation of the colon could have twisted the hypermobile gallbladder, resulting in torsion [3]. Sigmoid and cecal volvulus following colonoscopy were also reported in patients with redundant colon and absence of normal fixation of the bowel to its mesentery, predisposing the formation of loops and volvulus [4, 5].

Looping of the endoscope is one of the most common challenges encountered during colonoscopy. A study by Shah *et al.* reported ~91% of looping during colonoscopy, with the ‘N-sigmoid’ loop being the most common [6]. Looping can result in the loss of control of the endoscope, patient discomfort and increase in rates of incomplete colonoscopy [7]. Various manoeuvres are employed to counteract looping, such as the application of abdominal compression [8].

**Figure 3:**
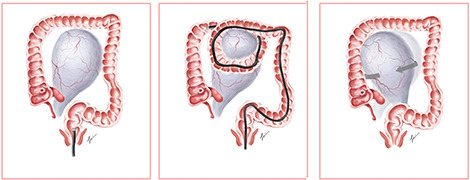
Illustration of looping precipitating ovarian torsion.

We propose a postulated mechanism of ovarian torsion in this patient (Fig. 3). Due to the large size of the ovarian mass, looping during colonoscopy could have caught the ovarian mass between the bowels, leading to the twisting of the mass on its pedicle by the looped segment of the bowel. Application of abdominal compression to advance the endoscope could have precipitated further twisting of the mass, resulting in ovarian torsion. Although causality between colonoscopy and ovarian torsion cannot be proven, the development of acute abdominal pain shortly after colonoscopy prompts us to consider that the colonoscope could have mechanically twisted the ovary on its pedicle.

To our knowledge, this is the first reported case of ovarian torsion following colonoscopy. Gastrointestinal evaluation in patients presenting with ovarian masses suspicious for malignancy is of utmost importance in view of the prevalence of ovarian metastases from the gastrointestinal tract. Nevertheless, we should always conduct these evaluations with heightened care, especially since the anatomy is altered in this group of patients. When counselling for complications of colonoscopy, the patient should be aware of ovarian torsion as a possible complication. Loops should be avoided during the procedure. While a variety of manoeuvres to counteract looping have been described [9], perhaps one should consider avoiding abdominal pressure in view of the postulated mechanism of torsion of large ovarian masses. Other techniques such as minimizing sedation may be considered, as abdominal discomfort during the scope could provide clues to the endoscopist that loops are encountered.

In patients with acute abdomen pain following endoscopy, perforation is often the foremost on an endoscopist’s mind. However, chest X-ray may not reveal free air. Clinical examination should be performed to check for peritonitis. Clinicians should be cognizant of acute ovarian torsion as a cause of severe abdominal pain following endoscopy.

## FUNDING

CAJO is funded by the National Medical Research Council Transition Award (NMRC/TA/0061/2017).

## CONFLICT OF INTEREST STATEMENT

The authors declare that they have no conflicts of interest.

## References

[1] Agnes A, Biondi A, Ricci R, Gallotta V, D'Ugo D, Persiani R. Krukenberg tumors: seed, route and soil. Surg Oncol 2017;26:438–45. 29113663 10.1016/j.suronc.2017.09.001

[2] Reumkens A, Rondagh EJ, Bakker CM, Winkens B, Masclee AA, Sanduleanu S. Post-colonoscopy complications: a systematic review, time trends, and meta-analysis of population-based studies. Am J Gastroenterol 2016;111:1092–101. 27296945 10.1038/ajg.2016.234

[3] Warfe SR, Dobson H, Hong MK, Ranasinghe WK, Thomas PR, Cichowitz AG. Torsion of wandering gallbladder following colonoscopy. Case Rep Med. 2013;2013:808751. 23956753 10.1155/2013/808751 PMC3730358

[4] Brown SR, Orgles CS, Lintott DJ, Finan PJ. Sigmoid volvulus after diagnostic endoscopy. Endoscopy 1997;29:50. 9083741 10.1055/s-2007-1004065

[5] Viney R, Fordan SV, Fisher WE, Ergun G. Cecal volvulus after colonoscopy. Am J Gastroenterol 2002;97:3211–2. 12492222 10.1111/j.1572-0241.2002.07144.x

[6] Shah SG, Saunders BP, Brooker JC, Williams CB. Magnetic imaging of colonoscopy: an audit of looping, accuracy and ancillary maneuvers. Gastrointest Endosc 2000;52:1–8. 10882954 10.1067/mge.2000.107296

[7] Witte TN, Enns R. The difficult colonoscopy. Can J Gastroenterol 2007;21:487–90. 17703247 10.1155/2007/520431 PMC2657972

[8] Waye JD, Yessayan SA, Lewis BS, Fabry TL. The technique of abdominal pressure in total colonoscopy. Gastrointest Endosc 1991;37:147–51. 2032597 10.1016/s0016-5107(91)70673-1

[9] Lee SH, Park YK, Lee DJ, Kim KM. Colonoscopy procedural skills and training for new beginners. World J Gastroenterol 2014;20:16984–95. 25493011 10.3748/wjg.v20.i45.16984 PMC4258567

